# A zoonotic human infection with simian malaria, *Plasmodium knowlesi*, in Central Kalimantan, Indonesia

**DOI:** 10.1186/s12936-016-1272-z

**Published:** 2016-04-16

**Authors:** Wuryantari Setiadi, Herawati Sudoyo, Hidayat Trimarsanto, Boy Adventus Sihite, Riahdo Juliarman Saragih, Rita Juliawaty, Suradi Wangsamuda, Puji Budi Setia Asih, Din Syafruddin

**Affiliations:** Eijkman Institute for Molecular Biology, Jakarta, Indonesia; Department of Parasitology, Faculty of Medicine, University of Hasanudin, Makasar, Indonesia; Muara Teweh Secondary Referral Hospital, Barito Utara Regency, Muara Teweh, Central Kalimantan Indonesia; Health Office of Central Kalimantan Province, Palangka Raya, Central Kalimantan Indonesia

**Keywords:** *Plasmodium knowlesi*, Mitochondrial *cytochrome c oxidase* sub-unit I (*mtCOI*), Central Kalimantan, Indonesia

## Abstract

**Background:**

The Indonesian archipelago is endemic for malaria. Although *Plasmodium falciparum* and *P. vivax* are the most common causes for malaria cases, *P. malariae* and *P. ovale* are also present in certain regions. Zoonotic case of malaria had just became the attention of public health communities after the Serawak study in 2004. However, zoonotic case in Indonesia is still under reported; only one published report of knowlesi malaria in South Kalimantan in 2010.

**Case presentation:**

A case of *Plasmodium knowlesi* infection in a worker from a charcoal mining company in Central Kalimantan, Indonesia was described. The worker suffered from fever following his visit to a lowland forest being cut and converted into a new mining location.

**Conclusion:**

This study confirmed a zoonotic infection using polymerase chain reaction amplification and Sanger sequencing of plasmodial DNA encoding the mitochondrial *cytochrome c oxidase* subunit I (*mtCOI*).

## Background

Endemic zoonotic malaria caused by *Plasmodium knowlesi* was confirmed in Malaysian Borneo in 2004. That discovery was not an outbreak, but corrected the misdiagnosis of such infections as *Plasmodium malariae*, despite an atypical hyper-parasitemia and far more severe clinical manifestations. Molecular analysis indeed found that the majority of malaria cases (120 out of 208 or 58 %) in Sarawak were caused by a primate malaria *P. knowlesi* [1]. Since then, *P. knowlesi* cases were also reported in Thailand [2–4], Phillippines [5], Singapore [6–8], Vietnam [9, 10] and Myanmar [11, 12]. In Indonesian Borneo (Kalimantan) that shares a border with the Malaysian state of Sarawak, a case of *P. knowlesi* in one patient was reported in 2010 [13]. As had occurred in Sarawak before 2004, malaria surveillance activities in Indonesia principally use microscopic diagnosis and no *P. knowlesi* infection was found among the surveyed areas. With the current deforestation activities, Indonesia is indeed very ripe for *P. knowlesi* risk [14].

In this report, a case of *P. knowlesi* malaria from a patient residing in Central Kalimantan was detected using *mtCOI* as a molecular target for a specific diagnosis. Mitochondrial *COI* gene has been used for diagnostic and species differentiation of a wide range of taxa [15]. The important advantage of *mtCOI* gene as marker for diagnosis and species differentiation is that it possesses a multiple copy number per haploid cell and a greater range of phylogenetic signal than any other mitochondrial and nuclear gene [16].

## Case presentation

**Fig. 1:**
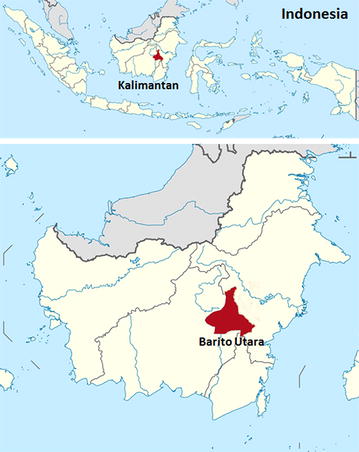
Map of Kalimantan, Indonesia, showing areas (*red*) where human *Plasmodium knowlesi* infection was found in Barito Utara, Central Kalimantan (obtained from http://commons.wikimedia.org/wiki/File: Lokasi Kalimantan Tengah Kabupaten Barito Utara.svg)

In September 2014, a 60-year old Indonesian male who worked at a coal mining company presented to hospital at Muara Teweh, Barito Utara regency, Central Kalimantan province (Fig. 1) with a 2-day history of fever, headache, musculoskeletal pain, and malaise. He was admitted to the hospital mentally alert, with a body temperature of 38.3 °C, blood pressure of 150/70 mm Hg, pulse rate 96/min, respiratory rate 24/min. Previously, the patient had spent 2 days in the jungle in Barito Utara District, Central Kalimantan surveying a new location for mining. Two weeks after returning from the jungle he experienced the onset of fever for 2 days and went to the hospital on the third day of fever.

On the first day of hospitalization, laboratory examination revealed mild anaemia, a normal leukocyte count, and slight thrombocytopaenia. There was an increase in the level of concurrent blood glucose, normal creatinine, and alanine aminotransferases. Urine analysis showed dark yellow and mild albuminuria (+1). The patient was also tested for typhoid fever, and the Widal test revealed no increase of antibody titres related to *Salmonella typhi* and *Salmonella paratyphi*. At day 2 of hospitalization, the blood laboratory profiles showed that there was a slight increase of haemoglobin level. The number of leukocytes was also increased, however thrombocyte decreased. Laboratory results are summarized in Table 1.

**Table 1:** Clinical assessment of the patient

Parameter	Values normal (range)		Values observed	
		Day 1 (admission)	Day 2	Day 5 (discharge)
Haemoglobin (g/dL)	[13.5–16]	11.6	12.4	11.0
Platelets (/µL)	[150,000–450,000]	120,000	50,000	200,000
Total white blood cells (/µL)	[4500–11,000]	8400	9500	8300
Basophils (%)	[0–1]	0	0	0
Eosinophils (%)	[0–5]	0	1	2
Neutrophils stab (%)	[0–5]	1	0	0
Neutrophils segment (%)	[50–70]	79	72	53
Lymphocyte (%)	[20–40]	20	27	41
Monocyte (%)	[1–6]	0	0	4
Random blood glucose (mg/dL)	[<140]	205	–	–
Total cholesterol (mg/dL)	[<200]	114	–	–
Creatinin (mg/dL)	[0.9–1.3]	1.2	–	1.5
Alanin amino transferase (IU/L)	[<41]	–	–	47
Aspartate amino transferase (IU/L)	[<40]	–	–	45
Widal Felix Test				
* Salmonella typhii* titer O		Negative		
* S. typhii* titer H		Negative		
* S. paratyphii* A titer O		1:160		
* S. paratyphii* A titer H		1:160		
* S. paratyphii* B titer O		Negative		
* S. paratyphii* B titer H		Negative		

**Fig. 2:**
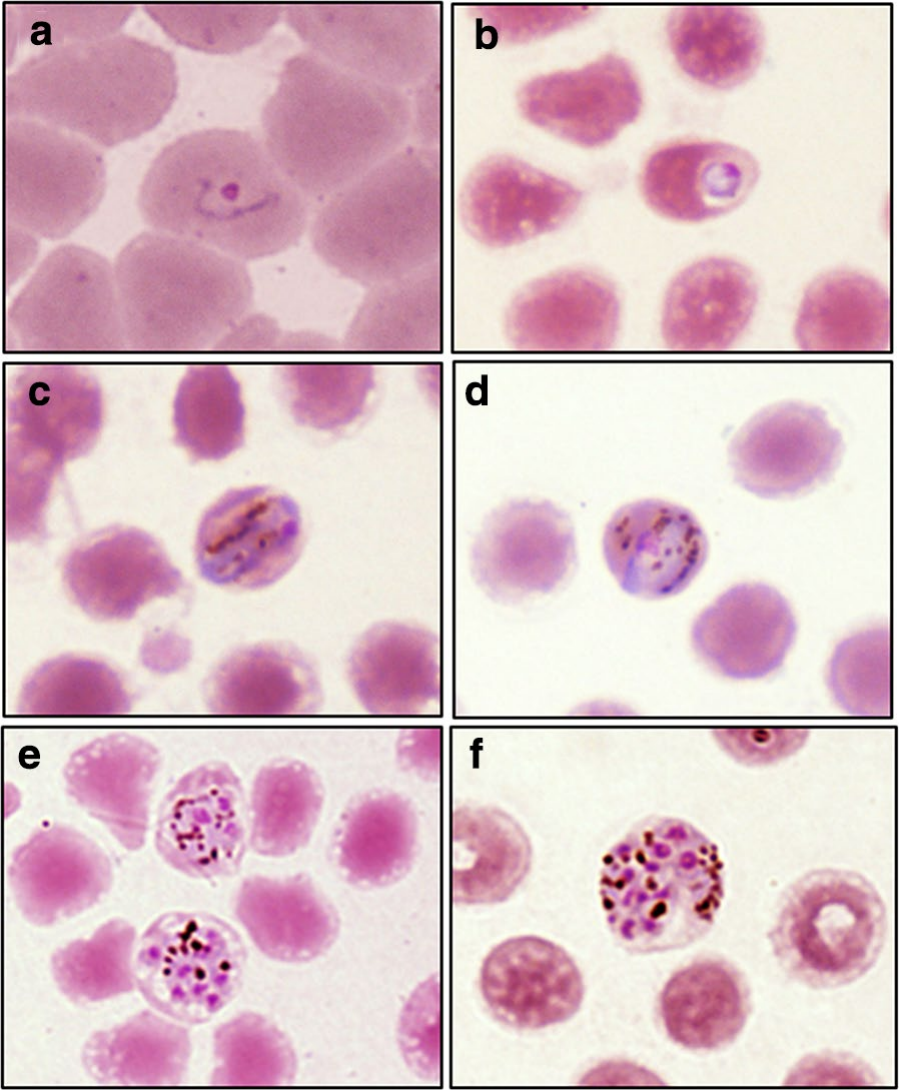
Morphology of *Plasmodium knowlesi* in a Giemsa-stained thin blood smear of subject in Barito Utara regency, Central Kalimantan showing a ring form (**a**, **b**), band form trophozoite (**c**, **d**), and schizonts (**e**, **f**)

Specific laboratory examination for malaria at the first day of admission to the hospital using a rapid diagnosis test (RDT) for malaria (Carestart Malaria HRP2/pLDH (Pf/PAN) Combo Access Bio Inc.) done in duplo. The first result was negative for both *Plasmodium falciparum* histidine rich protein 2 and aldolase, while the second result was slightly positive for pan-malarial aldolase antigen, but negative for *P. falciparum* histidine rich protein 2. That result of test prompted microscopic examination on Giemsa-stained thick and thin blood films that revealed a heavy parasitemia (1.25 % of red blood cells infected). The microscopist could definitively assign *Plasmodium* species identity to the infection. All stages of the parasites such as ring forms, trophozoites and schizonts were observed and the infected red blood cells were normal in size (Fig. 2).

The patient was treated with a standard oral regimen of dihydroartemisinin plus piperaquine plus 45 mg primaquine, intravenous crystalloid fluid 1500 ml/24 h, antipyretic and antihypertension drugs. Fever resolved within 24 h of initiating therapy and parasitemia had fallen to <0.01 %. By day 3 the patient had fully recovered and no parasites were detected by microscopy.

PCR analysis of the genomic DNA (gDNAs) extracted from the blood film was performed to determine the *Plasmodium* species. Firstly, the nested PCR assay was performed by using diagnostic primers for *Plasmodium* small sub-unit (SSU) rRNA as described [17], but were negative in the four human malaria parasite-specific PCR assays, including species specific primers for determining *P. knowlesi* [1].

Secondly, another PCR method targeting at the region of the *mtCOI* gene was performed followed by DNA sequencing of the amplicon. PCR amplification was performed by using primer pairs of PlasmoCOI-FDB 5′ ATACAAATTGTAATCATAAAACTTTAGG 3′ and PlasmoCOI-RDB 5′ ACTTCAGGATGTCCAAAAAACCA3′ in a total volume of 25 µL containing 10 ng/µl *Plasmodium* DNA, 10× PCR buffer (KAPA Biosystems, USA), 3 mM MgCl_2_ (KAPA Biosystems), 100 µM dNTPs (New England BioLabs, USA), 0.2 µM of each primer, of 80 µg/µL BSA (New England BioLabs) and 1.25 units of Taq DNA Polymerase (KAPA Biosystems). GeneAmp PCR system 9700 (Applied Biosystems, USA) was used to generate the following conditions: denaturation at 94 °C for 2 min, five cycles of 94 °C for 30 s, annealing at 56 °C for 45 s, and extension at 72 °C for 1 min, followed by 35 cycles of 95 °C for 30 s, 60 °C for 45 s, and 72 °C for 1 min, with a final extension at 72 °C for 5 min. This PCR method revealed DNA bands which was approximately 670 bp in length. The amplicons were then purified using a QIAquick PCR Purification Kit (Qiagen, Germany) and directly sequenced using the BigDyeTM Terminator v3.1 Cycle Sequencing Kit on a 3130xl Genetic Analyzer (Applied Biosystems). The DNA control samples originated from a known, archived *P. falciparum* and *Plasmodium vivax* gDNAs were used as a positive control.

**Fig. 3:**
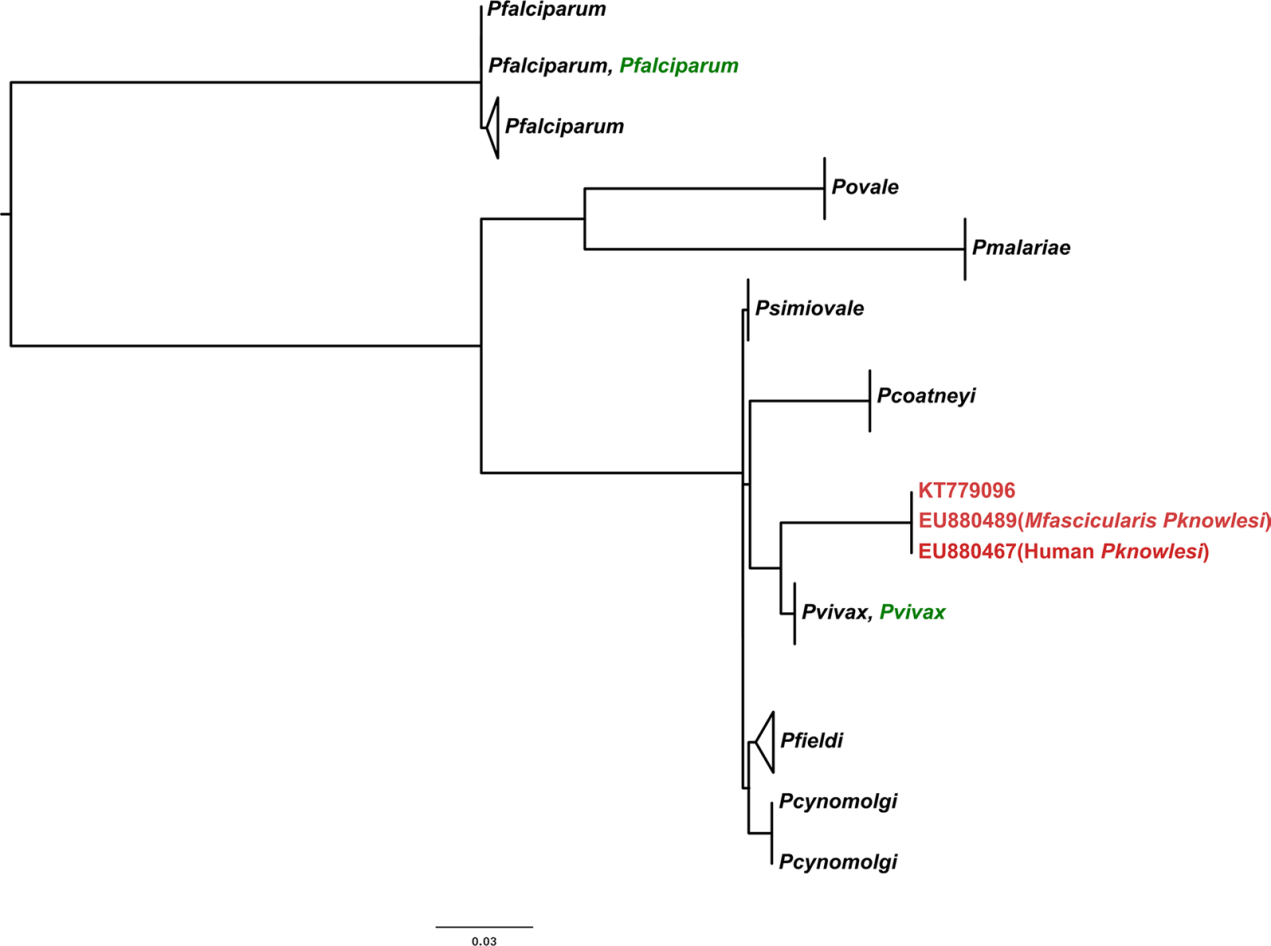
Maximum clade credibility phylogenetic tree from 670 bp COI-region of *Plasmodium* family obtained using MrBayes. The tree showed that the sequence of the patient (GenBank acc. no. KT779096) clustered strongly with *P. knowlesi* *mtCOI* sequence. The *Plasmodium vivax* and *Plasmodium falciparum* control positive were labelled with *green colour*

The 670 bp *mtCOI* fragment from the sample (PLK_ARB2146; GenBank acc. no. KT779096) had 100 % sequence identity with *P. knowlesi* sequences obtained from both *Macaca fascicularis* sample (Genebank acc. no. EU880489) and human samples (Genebank acc. no. EU880467, EU880464, EU880461, EU880460, EU880457, EU880451, EU880448) from other study [18]. Bayesian MCMC phylogenetic tree based on the *mtCOI* sequences of *Plasmodium* species showed that *mtCOI* sequences from both sample and other *P. knowlesi* sequences isolated from human and monkey, as obtained from Genbank, clustered together and formed a monophyletic clade, separated from other *Plasmodium* species clades. These sequence analysis confirmed that the patient was infected with the *P. knowlesi* parasite (Fig. 3) [19].

To further confirm of the result, a nested PCR assay was performed using the *mitochondrial cytochromeb* gene (*cytb*) as described [20, 21] and followed by sequencing analysis. Primers for the primary PCR were *Plasmodium* genus-specific and those for secondary PCR were specific for *P. knowlesi*. Forward primers for the primary and secondary PCRs were as reported by Putaporntip et al. [20] and the reverse primers were as reported by Tanizaki et al. [21]. The PCR yield an expected amplicon size of 130 bp and the sequencing results displayed 100 % identity with that of *P. knowlesicytb* gene (EU880498.1) [22].

Seventy-five days after the patient was hospitalized, a mass blood survey was carried out of 138 workers, including the former patient, at the coal mining company where the patient routinely worked. The mass blood survey consisted of microscopic examination and molecular method using nested PCR assay for *Plasmodium* 18S-rRNA gene and PCR sequencing of *mtCOI* gene. Both the microscopic examination and the nested PCR assay for 18S-rRNA gene revealed no infections by any malaria parasite species for all subjects. The PCR-sequencing assay using the *mtCOI* gene negative for all subjects, with the exception of the former patient which remained positive for *P. knowlesi* and reconfirmed by *cytb* gene sequencing indicating that he was apparently asymptomatic and microscopically sub-patent.

The failure to detect the parasites using the other PCR method that target the 18S rRNA gene might be related to the very limited gDNAs obtained through extraction using Giemsa-stained thick blood smear- the parasite density of the blood slide is 1.25 %. This reason was also supported when the PCRs were conducted on during the mass blood survey in which the gDNAs was extracted directly from the whole blood and all are negative by microscopy and only found positive for the *mtCOI* PCR. The mitochondrial DNAs of the malarial parasite are usually multi-copies (20-150 copies) in haploid cell whereas the 18S rRNA gene has small copy number (two-seven copies) per cell [23, 24]. A low target copy number limits the capabilities of detection, if the parasitemia is low.

The overall results indicated that either the standard malaria treatment using dihydroartemisinin-piperaquine and primaquine did not cure completely the patient or the subject may have been re-infected. However, the patient history of no traveling to the jungle or another place after the treatment rather supports for the recrudescence case.

## Conclusions

Clearing of natural rainforest on the island of Borneo for agricultural and mining uses has greatly reduced the natural habitat of the non-human primates occurring there. These activities create ecological circumstances that bring humans into contact with malarial parasites and their natural mosquito vectors not normally encountered. The situation creates greatly amplified risk of disease transmission among the human and non-human primates of the region, both zoonotic and anthroponotic [25, 26].

The gold standard of malaria diagnosis has been microscopic examination for over 120 years, despite obvious shortcomings such as *P. knowlesi* being readily confused for *P. falciparum* or *P. malariae* even by experienced microscopists [27]. The current molecular method using SSU-rRNA primers for detecting *P. knowlesi* such as pmk8 and pmkr9 also rendered inconsistent amplicon [9, 28], and the primers may cross-react with *P. vivax* gDNAs [29]. In this study, using DNA extracted from the Giemsa-stained thick smear on a routine microscopic slide, the amplicons of region of the *mtCOI* gene was obtained successfully using the *mtCOI* primers and the analysis of the *mtCOI* sequence identified the infecting malaria parasite as *P. knowlesi*.

This report confirms a second human infection by *P. knowlesi* in Indonesian Borneo [13]. This finding increases the probability that the *P. knowlesi* infection represents a significant public health problem, as it does in neighbouring Malaysian Borneo. This case also underlines the importance of conducting active malaria screening in the area using both microscopy and molecular diagnostics to monitor the emergence of *P. knowlesi* human infection and possible re-infection rates.

## Consent

Written informed consent was obtained from the patient for publication of this case report and accompanying images. This study received ethical approval from the Eijkman Institute Research Ethics Commission—EIREC no. 58/2013.
